# The Design and Evaluation of a Portable Extracorporeal Centrifugal Blood Pump

**DOI:** 10.3389/fphys.2021.766867

**Published:** 2021-10-14

**Authors:** Peng Wu, Wenjing Xiang, Chengke Yin, Shu Li

**Affiliations:** ^1^Artificial Organ Technology Laboratory, School of Mechanical and Electric Engineering, Soochow University, Suzhou, China; ^2^Institute for Medical Device Control, National Institutes for Food and Drug Control, Beijing, China

**Keywords:** centrifugal blood pump, portability, wearability, flow field, CFD

## Abstract

In recent years, blood pumps have become the bridge to heart transplantation for patients with heart failure. Portability and wearability of blood pumps should be considered to ensure patient satisfaction in everyday life. To date, the focus has been on the development of portable and wearable peripheral components, little attention has been paid to the portable and wearable performance of the blood pump itself. This study reported a novel design of a wearable and portable extracorporeal centrifugal blood pump. Based on an in-house centrifugal maglev blood pump, the wearable and portable blood pump was designed with parallel inlet and outlet pipes to improve the wearable performance. A ring cavity was set at the inlet to convert the circumferential velocity of the inlet pipe to an axial velocity. The hydraulic and hemolytic performance of the baseline and portable blood pumps were analyzed and compared. Compared with the baseline pump, the hydrodynamic and hemolytic performance of the portable pump has been maintained without serious degradation. The results of this study will improve the life quality of patients with heart failure, and enhance the clinical benefits of artificial heart.

## Introduction

For end-stage heart failure, mechanical circulatory support (MCS) devices, including artificial heart systems such as extracorporeal blood pumps have gradually became the bridge to heart transplantation (Magruder et al., 2017; Gaffey et al., 2018; Shahreyar et al., 2018; Rogers et al., 2019; Virani et al., 2021). As a life-saving medical device, the artificial heart system should be safer, lighter, more reliable, more portable, more wearable, and suitable for a wider range of patients-from infants to adults, males to females, and to ensure patient satisfaction in everyday life. To date, the focus has been on the development of portable and wearable peripheral components. For instance, wireless technology has been adopted to develop new transcutaneous energy transmission (TET) power systems (Rintoul and Dolgin, 2004). Compact wearable controller, and backup battery packs which are capable of being carried in a bag or by a small bedside monitor, or tied to the waist belt, have been suitably miniaturized as the wearable system (Vetter et al., 1995; Akdis and Reul, 2005). Portable driver and drive console were also developed to allow for the patient to be discharged home and facilitate ambulation of patients (Slaughter et al., 2007; Gregory et al., 2011).

Nonetheless, little attention has been paid to the portable and wearable performance of the blood pump itself through structural design. Volume displacement or pulsatile pumps were often designed with parallel inlet and outlet pipes to reduce overall size (Samuels et al., 2005; Berlin Heart AG, 2006; Zhang et al., 2007). To date, the majority of extracorporeal blood pumps are centrifugal pumps (Gregory et al., 2017). Their inlet and outlet pipes are perpendicular to each other. This significantly increases the length of the pipeline and takes up much space, which is a disadvantage to wearing and impedes the patients’ daily life and activities. Therefore, portable and wearable performance should be considered in the structural design process of the pump to increase the quality of life of patients.

Herein, we report a novel design of a portable extracorporeal centrifugal blood pump. Based on an in-house centrifugal maglev blood pump, the portable blood pump was designed with parallel inlet and outlet pipes to improve the wearable performance. A ring cavity was set at the inlet to convert the circumferential velocity of the inlet pipe to an axial velocity. The hydraulic and hemolytic performance of the baseline and portable blood pumps were analyzed and compared.

## Materials and Methods

### The Design of a Portable Blood Pump

**FIGURE 1:**
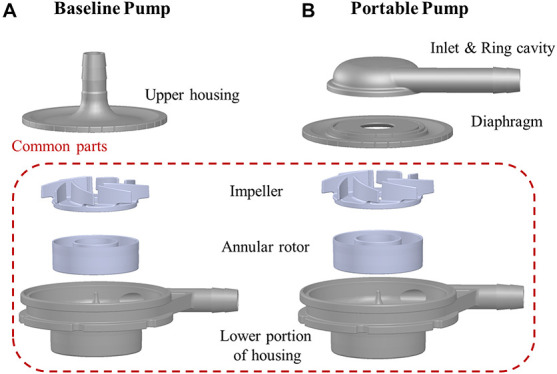
Exploded view of baseline blood pump **(A)** and portable blood pump **(B)**.

**FIGURE 2:**
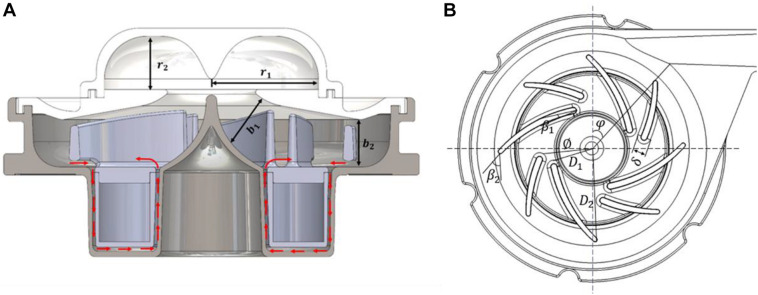
**(A)** Schematic of the portable blood pump. Red arrows indicate flow direction of the secondary flow path; **(B)** schematic of the impeller and volute, showing main design parameters.

The baseline design was based on a maglev blood pump previously developed in our group (Wu et al., 2021). The exploded view of the pump head is shown in Figure 1A. The magnetic rotor was combined with the rotor into an integral structure, and accommodated in a ring cavity. A secondary flow path was formed by the cavity and the lower portion of housing (cf. Figure 2A). The total blade number was eight, with four splitter blades. The baseline pump was designed using the speed coefficient method combined with the one-dimensional design theory (Wu et al., 2021). The design point was: 3,500 r/min, pressure head of 360 mmHg, and flow rate at 5 L/min. Figure 1B shows the portable blood pump. The portable blood pump shared the same impeller, annular rotor, and lower portion of housing with the baseline pump. The main difference is the upper portion, where the inlet is perpendicular to the pump axis and parallel to the outlet, a ring cavity was introduced to convert the circumferential inlet velocity to axial velocity. The main design parameters of the portable pump are shown in Figure 2 and Table 1.

**TABLE 1:** Parameters of the portable blood pump.

**Parameter**	**Symbol**	**Value**
Height of the ring cavity	*r*_1_	10 mm
Radius of ring cavity	*r*_2_	20 mm
Inlet diameter of impeller	*D*_1_	9.6 mm
Outlet diameter of impeller	*D*_2_	54 mm
Inlet width of blade	*b*_1_	7.3 mm
Outlet width of blade	*b*_2_	7 mm
Blade inlet angle	β_1_	14°
Blade outlet angle	β_2_	41.7°
Number of blades	Z	8

### Computational Fluid Dynamics Analysis

**FIGURE 3:**
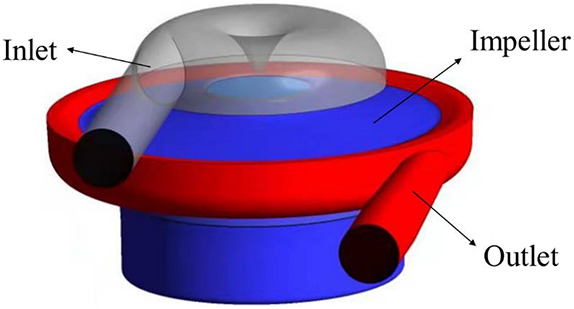
Schematic of computational domain for the portable blood pump.

Computational fluid dynamics (CFD) were employed to evaluate the hydraulic and hemolytic performance of both pumps. The grids were tetrahedral with five prism layers, generated using Ansys meshing (Ansys, Inc., Canonsburg, PA, United States). A cylindrical surface was placed downstream of the blade trailing edge, which acts as an interface between the rotating regions of the impeller and secondary flow path, and the region of volute and outlet. Similarly, another rotor/stator grid interface was place at the diaphragm, which separates the ring cavity and the region of impeller (as shown in Figure 3). A grid sensitivity analysis was also performed for the portable blood pump, with three grids of 4.58, 10.88, and 24.38 million, respectively (as shown in Table 2). The mean y^+^ was kept below 1 for the “middle” and “fine” grids. y^+^ may exceed 1 at some locations. To deal with this, the SST k-ω model was employed in this study, together with the wall function of “Enhanced wall treatment (EWT).” The EWT provides a unified empirical relationship for the buffer layer and log layer, and is y^+^ insensitive. The CFD simulations were performed using the commercial software Ansys Fluent. The flow rate of 5 L/min was applied at the inlet. The blood was regarded as a Newtonian fluid, and the viscosity was taken as 3.5 mPa s. A SIMPLE method was employed to solve the incompressible N-S equations. Turbulence was modeled using the RNG k-ε model. The steady “frame motion” approach was used to couple the rotating and stationary regions. Convergence criteria was set that the residuals of all equations drop below 10^–6^. The computational setup of the baseline blood pump is the same as those of the portable pump. The same computational setup has been employed in our previous study and verified through hydraulic experiments (Wu et al., 2021). For grid sensitivity analysis, the rotational speed was set as 3,500 rpm, and inlet flow rate was set as 5 L/min for the portable pump. The H-Q curves of both pump models were computed, with flow rates ranging from 1 to 9 L/min and pressure head ranging from 100 to 900 mmHg. A detailed analysis and comparison of hydrodynamic and hemolytic performance was conducted at the design point (5 L/min, 360 mmHg) for both pump models. For each pump model, the rotational speed of the rotor was adjusted through iterative computations to meet the targeted pump head of 360 mmHg.

**TABLE 2:** Details of mesh for grid sensitivity analysis.

**Mesh**	**Cells (×10^6^)**	**Mean y^+^ on impeller surface**
Coarse	4.58	1.42538
Middle	10.88	0.930659
Fine	24.38	0.765471

### Hemolysis Predictions

The energy-dissipation-based (EDB) formulation of pow-law models were employed in this study to estimate hemolysis level of the blood pumps. The EDB models relate hemolysis to energy dissipation ε and exposure time *t* through a power-law relationship (Giersiepen et al., 1990; Wu et al., 2019)


(1) $$HI\left(\%\right)=\frac{h\,b}{H\,b}\times 100=C{\left(\varepsilon \mu \rho \right)}^{\alpha /2}t^{\beta },$$

where *HI*(%) is the hemolysis index in percentage, ε is energy dissipation rate, *Hb* is the total hemoglobin concentration, *hb* represents the increase in plasma free hemoglobin; *C*, α, and β are empirical constants. Energy dissipation rate ε is calculated by adding up viscous dissipation rate ε_*vis*_ and turbulent dissipation rate ε_*turb*_. Please refer to Wu et al. (2019) for the definition of ε_*vis*_ and ε_*turb*_.

It has been shown that Reynolds stress is an inaccurate indicator of turbulent effects on hemolysis, and the stress-based formulation of power-law hemolysis models tend to over-predicted hemolysis (Jones, 1995; Hund et al., 2010; Wu et al., 2019, 2020). In contrast, the EDB formulation can improve the prediction of hemolysis for a wide range of flow conditions, especially turbulent flows (Wu et al., 2019, 2020). Three widely used sets of empirical constants were employed in this study. Table 3 lists the empirical constants of the three power-law models employed in this study. One can note that the exposure time in Eq. 1 is non-linear. It would be incorrect to consider that the hemolysis at the domain outlet is the sum of the local hemolysis, because of the non-linear dependency in time. This problem was avoided by introducing *hb*′ as a scalar variable equal to *hb*^1/β^. Then Eq. 1 can be reorganized into a Eulerian scalar transport equation (Garon and Farinas, 2004).


(2) $$\frac{d\,\left(h\,b^{\prime }\right)}{d\,t}+v\,\rho \cdot \nabla \left(h\,b^{\prime }\right)=C,$$

where *C* is the source term defined as


(3) $$C=\rho \,{\left(H\,b\cdot c\cdot {\left(\varepsilon \,\mu \,\rho \right)}^{\alpha /2}\right)}^{1/\beta },$$

The *HI*(%) was then calculated from the mass-weighted average of *hb* at the outlet of the device divided by *Hb*. Hemolysis predictions started after flow simulations had converged, with all flow variables frozen.

**TABLE 3:** Empirical constants of the stress-based power-law hemolysis models.

**Model**	**C**	α	β
GW	3.620 × 10^–5^	2.4160	0.7850
HO	1.800 × 10^–4^	1.9910	0.7650
TZ	1.228 × 10^–5^	1.9918	0.6606

*GW, model of Giersiepen et al. (1990). The constants of the HO model were originally derived from Heuser and Opitz (1980) by Song et al. (2003). TZ, model of Zhang et al. (2011).*

## Results

### Grid Sensitivity Analysis

The results of grid sensitivity analysis are shown in Table 4. Pressure head and hemolysis index were compared to the results predicted with the fine grid. The HO model was used for hemolysis prediction. For the “Middle” grid, the error of pressure head was within 2%, and the error of hemolysis was within 1%. Therefore, a grid of around 10 million is the most appropriate, with results sufficiently resolved and relatively low computational cost compared with the finer mesh. Therefore, this grid was chosen for all the simulations of portable blood pump. The grid of baseline pump was generate using the same setting as the “Middle” grid of portable pump, also with size of around 10 million.

**TABLE 4:** Results of grid sensitivity analysis for the portable pump, 5 L/min, 3,500 rpm.

**Mesh**	**Cells (×10^6^)**	**P (mmHg)**	**Error of P (%)**	**Error of HI (%)**
Coarse	4.58	390.6954	2.57	1.37
Middle	10.88	393.0736	1.72	0.41
Fine	24.38	399.9683	/	/

*P, predicted pressure head; error of P (%), defined as | P − P_0_| /P_0_, where P_0_ is the pressure head predicted with the fine mesh; HI (%), hemolysis predicted using the HO model; error of HI (%), defined as | HI − HI_0_| /HI_0_, where HI_0_ is the HI predicted with the fine mesh.*

### Hydrodynamic Performance

**FIGURE 4:**
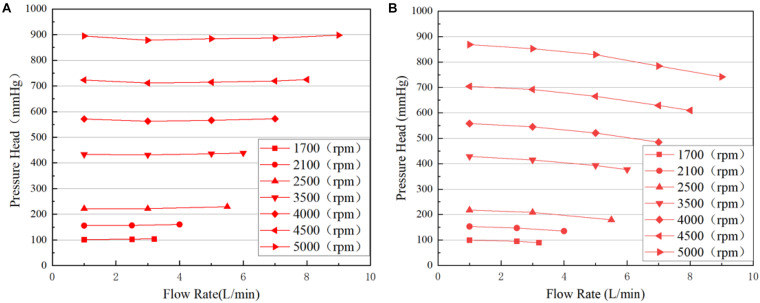
Predicted H-Q curves: **(A)** baseline blood pump; **(B)** portable blood pump.

The H-Q curves of both pump models predicted using CFD were shown in Figure 4. The H-Q curves of the baseline pump were very flat, and were almost constant as flow rate increases. This was probably caused by the cylindrical volute. At low flowrates, secondary flows and turbulence intensities were very high, bringing high flow loss. The pressure head of the portable blood pump decreases with the increase of Q. Lower pressure head at high flow rates for portable pump means more flow losses, which might be caused by the ring cavity. This will be further analyzed in the following sections.

### Pressure and Flow Patterns

**FIGURE 5:**
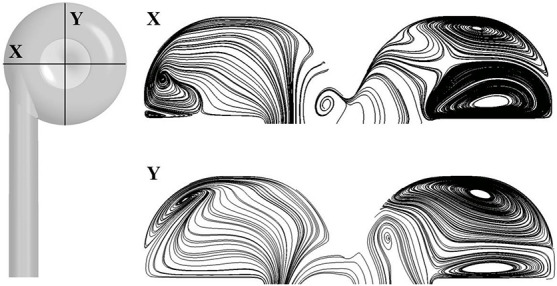
Surface streamlines at two cross-sections in the ring cavity.

We focus on the pressure and flow field at the design point of 5 L/min, 360 mmHg. Table 5 shows the pressure head in different portions of the two pump models (as shown in Figure 3). Since steady simulations were conducted, total pressure was mass-weighted averaged at the inlet, outlet and interfaces of different portions. Pressure head of different portions were then calculated by subtracting the total pressure at the downstream interface/outlet from the upstream interface/inlet. The corresponding rotational speeds of the baseline and portable pumps were 3,160 and 3,350 rpm, respectively. The hydraulic efficiency of the baseline pump is around 3% higher than the portable pump. To achieve the same pressure head, the rotational speed of the portable pump was higher than the baseline pump. The pressure loss in the inlet (including ring cavity) portion of the portable pump is considerable, while the pressure loss is negligible in the inlet portion of the baseline pump. Figure 5 shows the streamlines at two cross-sections in the ring cavity, in which large vortices were clearly visible. The likely reasons for the formation of vortices in the ring cavity are the expansion of the flow path and the Coriolis force.

**TABLE 5:** Pressure head in different portions of the two pump models, in mmHg.

**Pump**	**Speed (rpm)**	**Inlet**	**Impeller**	**Outlet**	**Total**	**Hydraulic efficiency (%)**
Baseline	3,160	−0.93	740.37	−380.85	358.58	19.87
Portable	3,350	−27.89	761.65	−371.83	361.93	16.79

**FIGURE 6:**
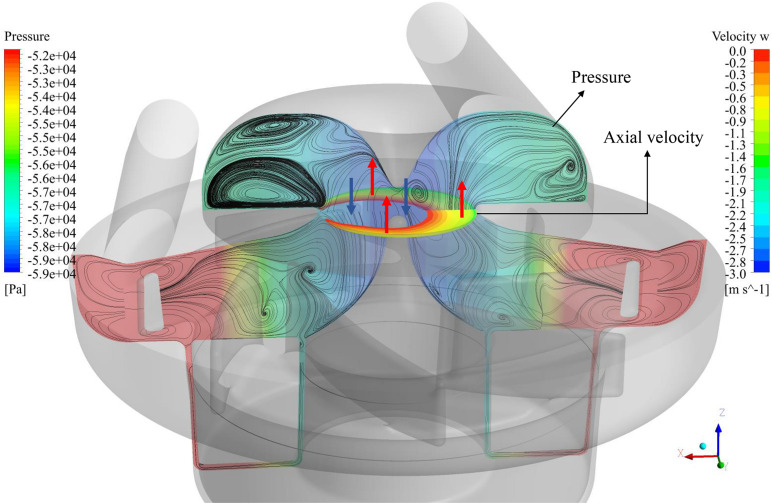
Flow patterns at the meridional plane, where surface streamlines and pressure contours are shown. Positive axial velocity (backflow) contours at the cavity/impeller interface are also shown.

Figure 6 shows the flow patterns at the meridional plane, where surface streamlines and pressure contours are shown. Low-pressure zone exists at the center of ring cavity. The contours of positive axial velocity at the ring cavity/impeller interface are also shown in Figure 6. Positive axial velocity means backflow, happening at the outer area of the interface (as shown by the red arrows). The backflow is most likely caused by the low-pressure zone at the center of ring cavity.

**FIGURE 7:**
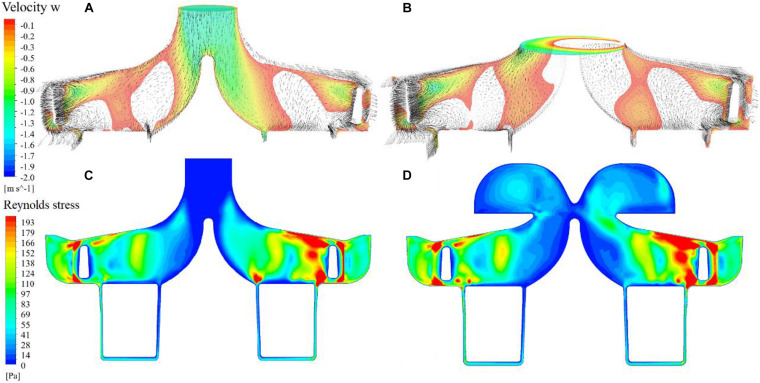
Negative axial velocity contours at the meridional plane and cavity/impeller interface for the baseline and portable pumps **(A,B)**; contours of Reynolds stress at the meridional plane for the baseline and portable pumps **(C,D)**.

Figures 7A,B show negative axial velocity contours at the meridional plane for both pump models. Regions of backflow can be observed at the entrance of the impeller. Figures 7C,D show contours of Reynolds stress. The Reynolds stress in the ring cavity of the portable pump is fairly high, reflecting the influence of vortices. On the other hand, the regions of backflow are also regions where Reynolds stress is low, showing that backflow reduced turbulence at the impeller. This explains that the pressure head of the impeller for the portable pump is higher than the baseline pump.

### Hemolysis

As aforementioned, three sets of empirical constants were employed to estimate hemolysis. These empirical constants were widely used and each has its own advantages in accuracy in different situations. In order to minimize the uncertainty brought by hemolysis model, a new variable, *HI*_*diff*_, was introduced to represent the overall change of hemolysis over the baseline pump considering the results of all the three sets of empirical constants, defined as:


(4) $$H\,I_{d\,i\,f\,f}=\left(\frac{H\,I_{G\,W}^{\prime }}{H\,I_{G\,W}}+\frac{H\,I_{H\,O}^{\prime }}{H\,I_{H\,O}}+\frac{H\,I_{T\,Z}^{\prime }}{H\,I_{T\,Z}}\right)/3-1,$$

where the denominators represent the predicted hemolysis indices of the baseline pump, while primes represent the hemolysis indices of the portable blood pump. Subscripts “GW,” “HO,” and “TZ” refer to the hemolysis calculated using the three sets of empirical constants as shown in Table 3. At the design point (5 L/min, 360 mmHg), the value of *HI*_*diff*_ is 29%, i.e., the hemolysis level of the portable blood pump is 29% higher compared to the baseline pump.

## Discussion

In this study, a novel concept of portable centrifugal blood pump was proposed. The inlet pipe is parallel to the outlet pipe, to increase portability and wearability. There have been other extracorporeal pumps with the design of parallel inlet and outlet, such as Berlin Heart EXCOR. Nonetheless, these pumps are not rotary blood pumps. Centrifugal pumps are mainstream of both intracorporeal and extracorporeal blood pumps. For intracorporeal blood pumps, perpendicular inlet and outlet of centrifugal pumps are compatible with the way how they are installed. In contrast, for extracorporeal pumps, non-parallel inlet and outlet will hamper their wearability and portability.

This study represents the first attempt to improve the wearability of centrifugal blood pumps through structural design of the flow path. The design of the portable pump was based on an in-house centrifugal maglev blood pump which has been well-verified through experiments. The inlet and outlet pipes are parallel. A ring cavity was designed to convert the circumferential inlet velocity to an axial velocity. Numerical simulations were carried out to evaluate the hydrodynamic and hemolytic performance, with a focus on the condition of design point. Compared with the baseline pump, the hydrodynamic and hemolytic performance of the portable pump has been maintained without serious degradation, with the hydraulic efficiency dropped by ∼3%, and hemolysis index increased by 29%. The main cause of degradation in performance is the ring cavity, where strong secondary flows happened, bringing flow loss and extra damage to the blood.

This study has several limitations. Firstly, this study merely conducted a preliminary design of the portable centrifugal blood pump. The ring cavity caused considerable flow loss and blood damage. Thus, optimization will be needed considering multiple geometric parameters, to reduce the secondary flows in the cavity. Secondly, although the computational setup has been previously verified with hydraulic experiments of the baseline pump, since the flows are more chaotic in the portable blood pump, experimental verification will still be needed to verify the accuracy of CFD results. Finally, although EDB formulation of hemolysis models were employed together with three commonly used sets of empirical constants, to minimize errors in hemolysis prediction, hemolysis tests will still be required to validate the hemolytic performance of the portable blood pump.

## Data Availability Statement

The original contributions presented in the study are included in the article/supplementary material, further inquiries can be directed to the corresponding authors.

## Author Contributions

PW and CY: study concept and design. WX and PW: numerical simulation and data analysis, and drafting of the manuscript. PW, CY, and SL: critical revision of the manuscript. All authors contributed to the article and approved the submitted version.

## Conflict of Interest

The authors declare that the research was conducted in the absence of any commercial or financial relationships that could be construed as a potential conflict of interest.

## Publisher’s Note

All claims expressed in this article are solely those of the authors and do not necessarily represent those of their affiliated organizations, or those of the publisher, the editors and the reviewers. Any product that may be evaluated in this article, or claim that may be made by its manufacturer, is not guaranteed or endorsed by the publisher.
